# Nasogastric Nutrition versus Nasojejunal Nutrition in Patients with Severe Acute Pancreatitis: A Meta-Analysis of Randomized Controlled Trials

**DOI:** 10.1155/2016/6430632

**Published:** 2016-06-02

**Authors:** Youfeng Zhu, Haiyan Yin, Rui Zhang, Xiaoling Ye, Jianrui Wei

**Affiliations:** ^1^Department of Intensive Care Unit, Guangzhou Red Cross Hospital, Medical College, Jinan University, Guangzhou 510220, China; ^2^Institute of Clinical Nutrition, Guangzhou Red Cross Hospital, Medical College, Jinan University, Guangzhou 510220, China

## Abstract

*Introduction.* Previous studies have shown that the nasogastric (NG) route seems equivalent to the nasojejunal (NJ) route in patients with severe acute pancreatitis (SAP). However, these studies used a small sample size and old criteria for diagnosing SAP, which may include some patients with moderate SAP, according to the newly established SAP criteria (Atlanta 2012 classification). Based on the changes in the criteria for classifying SAP, we performed an up-to-date meta-analysis.* Method.* We reviewed the PubMed, EMbase, China National Knowledge Infrastructure, Wanfang Database, and Cochrane Central Register of Controlled Trials electronic databases. We included randomized controlled trials comparing NG and NJ nutrition in patients with SAP. We performed the meta-analysis using the Cochrane Collaborations' RevMan 5.3 software.* Results.* We included four randomized controlled trials involving 237 patients with SAP. There were no significant differences in the incidence of mortality, infectious complications, digestive complications, achievement of energy balance, or length of hospital stay between the NG and NJ nutrition groups.* Conclusions.* NG nutrition was as safe and effective as NJ nutrition in patients with SAP. Further studies are needed to confirm our results.

## 1. Introduction

Acute pancreatitis is one of the most common diseases of the digestive system, leading to large physical and economic burdens [1, 2]. Recent studies [3, 4] indicate that the incidence of acute pancreatitis varies between 4.9 and 73.4 per 100,000 worldwide.

Severe acute pancreatitis (SAP) occurs in 15%–20% of acute pancreatitis patients [5] and is characterized by a high mortality rate. It is a potentially fatal disease that requires nutritional support [6, 7], which is considered a primary issue in the therapy of the disease, as well as a secondary issue in addressing extended pancreatic and extrapancreatic inflammation [6].

Multiple randomized trials [8–10] have suggested that enteral nutrition (EN) is associated with an increased capacity of the intestinal mucosal barrier and a decrease in infectious complications in patients with SAP because EN maintains the mucosal barrier of the gut, protects the intestinal mucosa, and prevents the translocation of the bacteria that cause pancreatic necrosis [9, 10]. Therefore, nutritional support using EN in patients with SAP has been recommended by many acute pancreatitis guidelines [5, 11–13].

Many studies have shown that NJ nutrition is an effective method of providing EN for patients with SAP [14]. NG has been believed to stimulate pancreatic secretion, causing an exacerbation of the inflammatory process in the pancreas [15, 16]. Moreover, NG nutrition may increase the risk of developing aspiration pneumonia [17]. Therefore, the NJ route is traditionally preferred to avoid the gastric phase of stimulation.

However, during the past decade there have also many successful studies that used NG nutrition in patients with SAP [18–20]. Some meta-analyses comparing NG nutrition with NJ nutrition found that NG nutrition was safe and could be well tolerated in patients with SAP [14, 21]. In those studies or meta-analyses, the criteria for diagnosing SAP were provided in the Atlanta 1993 classification. However, the criteria for diagnosing SAP have changed over time. According to the newest criteria of the Atlanta 2012 classification, SAP is defined by the presence of persistent (fails to resolve within 48 h) organ failure and/or death [22], which is different from the criteria of the Atlanta 1993 classification. Local complications (including pancreatic necrosis and/or transient organ failure (<48 h)), which were considered SAP in the Atlanta 1993 classification, were excluded from the Atlanta 2012 classification and were considered as moderately SAP. Therefore, the definition of SAP in the Atlanta 2012 classification was stricter than the Atlanta 1993 classification.

According to the guidelines for acute pancreatitis from the American College of Gastroenterology, in mild acute pancreatitis, oral intake is usually restored quickly, and no nutritional intervention is needed [5]. Mortality in patients with mild acute pancreatitis is usually rare. Additionally, according to the revised Atlanta classification, moderately severe acute pancreatitis has a lower mortality and requires less intervention than severe acute pancreatitis [11]. Therefore, we conclude that there may be no significant benefit of nasojejunal nutrition in mild and moderately severe acute pancreatitis. Nasojejunal nutrition may be helpful only in patients with SAP.

In those previous studies, some patients who had moderately SAP may have been considered as having SAP, and the results of the comparison between NG nutrition and NJ nutrition may be biased. As a matter of fact, some controversial therapies may only show efficacy in more critically ill patients.

Based on the changes in the classification criteria for SAP, we performed an up-to-date meta-analysis to compare the differences in the clinical outcomes of patients with SAP who received NG or NJ.

## 2. Materials and Methods

### 2.1. Data Sources and Search Strategy

We reviewed studies published in the Pubmed, EMbase, China National Knowledge Infrastructure, Wanfang Database, and Cochrane Central Register of Controlled Trials electronic databases. To identify the relevant studies, we also searched the references from the relevant articles. The keywords used for the searches were “severe acute pancreatitis” and “nasogastric or nasojejunal” and “nutrition or feeding” in different combinations, with limitations to randomized controlled trials. No limits on language, sample size, gender, or the location of the original study were entered for the search.

### 2.2. Study Selection

We determined the publications that were suitable for the meta-analysis using the following selection criteria: (1) randomized controlled trial (RCT); (2) population: hospitalized patients with SAP; (3) comparison between NG nutrition and NJ nutrition; and (4) evaluation of mortality. We used several outcome variables. The primary outcome was overall mortality, and the secondary outcome was at least one of the following variables: incidence of complications (tracheal aspiration, infection, diarrhea, or exacerbation of pain), achievement of energy balance, and length of hospital stay. All analyses were based on previously published studies; thus, ethical approval and patient consent are not required.

### 2.3. Data Extraction and Quality Assessment

Two independent reviewers (XiaoLing Ye and Rui Zhang) screened the titles and abstracts using a structured data abstraction form, which resulted in high and satisfactory interobserver agreement. Any disagreement was resolved by consensus or by consulting a third author (Jianrui Wei). We extracted the authors' names, title of the article, journal in which the study was published, country and year of the study, methodological variables, and clinical outcomes. The modified Jadad score was used to evaluate the quality of the included trials [23]. Two independent reviewers (Youfeng Zhu and Haiyan Yin) assessed the bias of the included studies according to the methods described in the Cochrane Handbook for Systematic Reviews of Interventions [24]. The following parameters were assessed: random sequence generation, blinding of participants and personnel, allocation concealment, blinding of outcome assessment, incomplete outcome data, and selective outcome reporting. According to the Cochrane Handbook, other sources of bias were a risk of bias related to the specific trial design used or the early termination of the study due to an extreme baseline imbalance in the selected patients.

### 2.4. Statistical Analysis

The Cochrane Collaboration's Review Manager Software 5.3 (RevMan 5.3) was used for the meta-analysis. The results were obtained by direct extraction or by indirect calculation. The risk ratios (RR) and 95% confidence intervals (CI) were calculated for the binary data, and the standardized mean differences (SMD) and 95% CI were calculated for the continuous data variables. Heterogeneity between trials was tested using the chi-square test, with *P* < 0.05 and *I* ^2^ greater than 50% indicating significant heterogeneity (difference). The random effects model was used to calculate the outcomes of both the binary and continuous variables, regardless of statistical heterogeneity. We used forest plots to graphically display the results. A funnel plot was used to uncover potential publication bias.

## 3. Results

**Figure 1:**
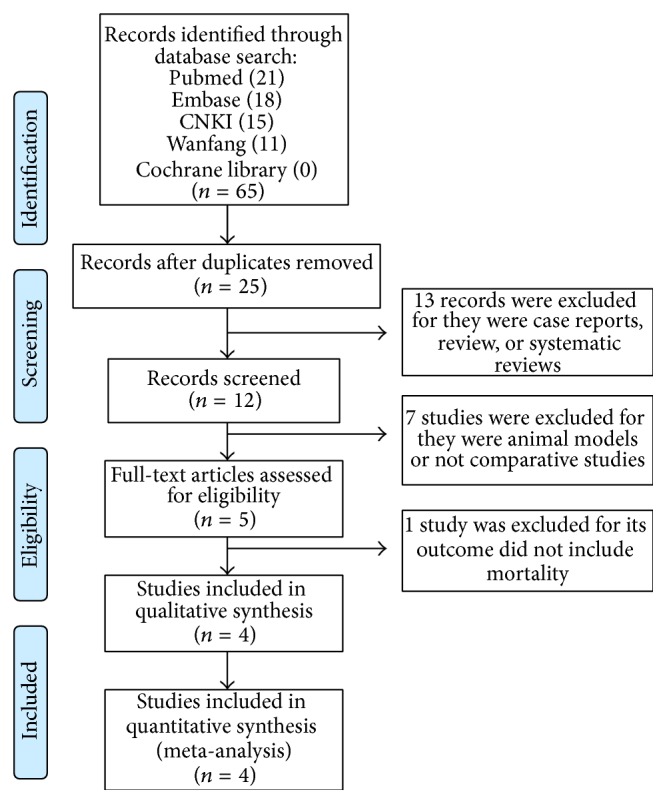
PRISMA flow chart for the study selection process.

Figure 1 shows the selection process for the eligible trials. First, 65 records were identified, including 21 records from PubMed, 18 records form EMbase, 15 records from China National Knowledge Infrastructure, and 11 records from Wanfang Database. After removing 40 duplicate records and 20 case-only studies, review articles, comments, or case reports, 5 records remained for assessment. One study was excluded due to insufficient data. Finally, 4 studies were included in the present meta-analysis [18, 19, 21, 25]. The characteristics and quality of the included RCTs are shown in Table 1.

A total of 237 patients with SAP were enrolled in the present study. Of these, 122 were randomly assigned to an NG group and 115 to an NJ group. The basic demographic characteristics of all patients are shown in Table 2.

**Figure 2:**
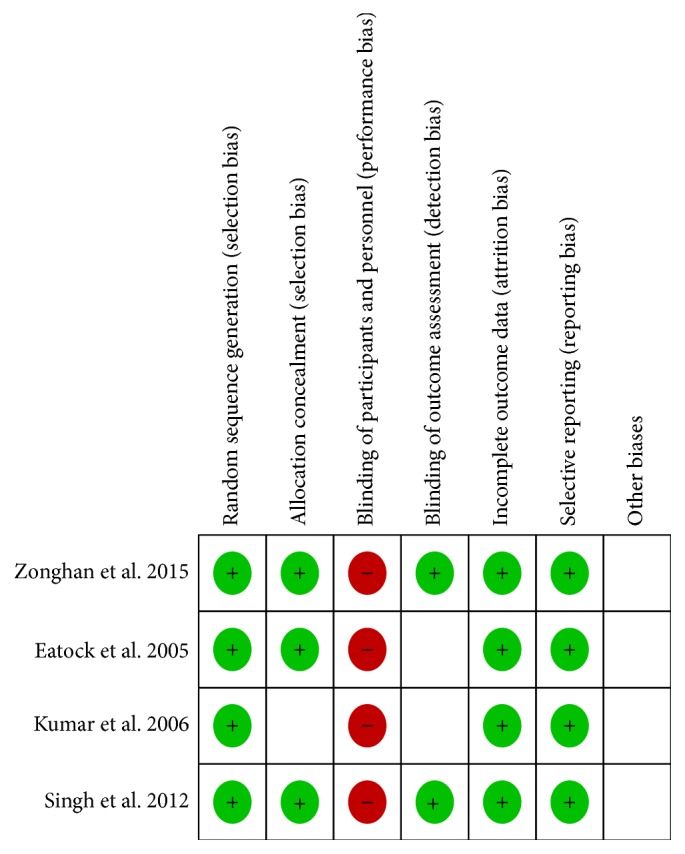
Risk of bias summary: reviewing authors' judgements about the risk of bias for each item in each included study.

*Risk of Bias in the Included Studies*. We used a tool from the Cochrane Collaboration to assess the risk of bias of each study and present the details of the results in Figure 2. Based on the nature of the study, we did not double blind the 4 studies; we believe that this did not influence the outcomes of the study.

### 3.1. Mortality

**Figure 3:**
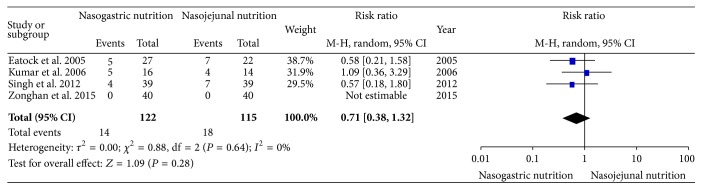
Comparison of mortality between the NG and NJ nutrition groups.

All of the included RCTs reported the mortality. There was no significant heterogeneity (*χ* ^2^ = 0.88, df = 2, and *P* = 0.64; *I* ^2^ = 0%) among the four studies. In the random effects model, there was no significant difference in the incidence of mortality between the NG and NJ groups (RR, 0.71; 95% CI, 0.38 and 1.32; *z* = 1.09 and *P* = 0.28, Figure 3).

### 3.2. Infectious Complications

**Figure 4:**
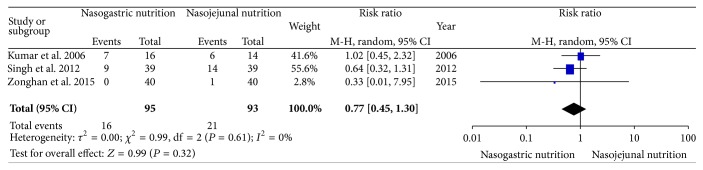
Comparison of the infectious complications between the NG and NJ nutrition groups.

With the exception of the study by Eatock et al., all three of the other included RCTs reported the infectious complications (blood culture, tracheal aspiration, bile culture, or pancreatic aspirate culture). In the NJ nutrition group of the study by Kumar et al., 2 patients were blood culture-positive, 1 patient was tracheal aspirate culture-positive, and 3 patients were pancreatic aspirate culture-positive; in the NG nutrition group, 3 patients were blood culture-positive, 3 patients were pancreatic aspirate culture-positive, and 1 patient was bile culture-positive. In the study by Singh, 9 and 14 patients were blood/tracheal aspirate/bile/pancreatic aspirate culture-positive in the NG and NJ nutrition groups, respectively. In the study by Du et al., no infectious complications were observed in the NG nutrition group, and 1 patient with pulmonary infection was observed in the NJ nutrition group. There was no significant heterogeneity (*χ* ^2^ = 0.99, df = 2, and *P* = 0.61; *I* ^2^ = 0%) among the three trials. In the random effects model, the risk of developing complications was similar in the NG group compared with the NJ group (RR, 0.77; 95% CI, 0.45 and 1.30; *z* = 0.99 and *P* = 0.32, Figure 4).

### 3.3. Digestive Complications

**Figure 5:**
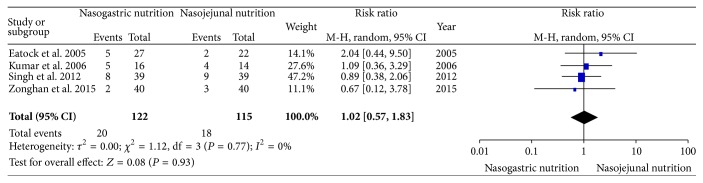
Comparison of the digestive complications between the NG and NJ nutrition groups.

All of the included studies reported digestive complications (abdominal bloating, diarrhea, or pain upon refeeding). In the NJ nutrition group of the study by Eatock et al., 1 patient with abdominal bloating and 1 patient with diarrhea were observed, and 3 patients with diarrhea and 2 patients with pain upon refeeding were observed in the NG nutrition group. In the study by Kumar, only 1 patient each in the 2 groups had a recurrence of pain, and diarrhea was observed in 4 and 3 patients in the NG and NJ nutrition groups, respectively. In the study by Singh, 1 patient with abdominal bloating, 4 patients with diarrhea, and 3 patients with pain upon refeeding were observed in the NG nutrition group; 1 patient with abdominal bloating, 3 patients with diarrhea, and 5 patients with pain upon refeeding were observed in the NJ nutrition group. In the study by Du et al., digestive complications were observed in 2 and 3 patients in the NG and NJ nutrition groups, respectively; however, the data regarding the types of complications were unclear. There was no significant heterogeneity (*χ* ^2^ = 1.12, df = 3, and *P* = 0.77; *I* ^2^ = 0%) among the four trials. In the random effects model, there was no significant difference in the digestive complications between the NG and NJ nutrition groups (RR, 1.02; 95% CI, 0.57 and 1.83; *z* = 0.08 and *P* = 0.93, Figure 5).

### 3.4. Achievement of Energy Balance

**Figure 6:**
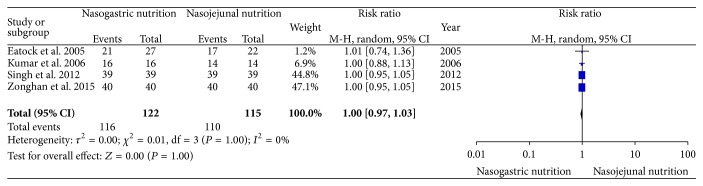
Comparison of the achievement of energy balance between the NG and NJ nutrition groups.

All of the enrolled studies reported the achievement of energy balance. There was no significant heterogeneity (*χ* ^2^ = 0.01, df = 3, and *P* = 1.00; *I* ^2^ = 0%) among the four trials. In the random effects model, there was no significant difference in the achievement of energy balance between the NG and NJ nutrition groups (RR, 1.00; 95% CI, 0.97 and 1.03; *z* = 0.00 and *P* = 1.00, Figure 6).

### 3.5. Length of Hospital Stay

**Figure 7:**
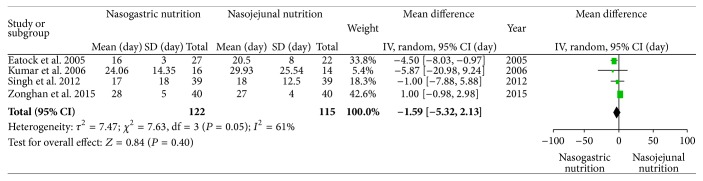
Comparison of the lengths of hospital stay between the NG nutrition and NJ nutrition groups.

All four studies reported the length of hospital stay. In the study by Eatock et al., the lengths of hospital stay were 16 (range 10–22 days) and 15 (range 10–42 days) days in the NG and NJ nutrition groups, respectively. In the study by Singh, the lengths of hospital stay were 17 (range 1–73 days) and 18 (range 4–54 days) days in the NG and NJ nutrition groups, respectively. We recalculated these data and converted them into means and standard deviations [26]. There was significant heterogeneity (*χ* ^2^ = 7.63, df = 3, and *P* = 0.05; *I* ^2^ = 61%) among the studies. There were no significant differences between the NG and NJ groups (SMD −1.59; 95% CI, −5.32 and 2.13; *z* = 0.84 and *P* = 0.40, Figure 7). There were several causes of heterogeneity, such as the nation of origin and the economic development level.

**Figure 8:**
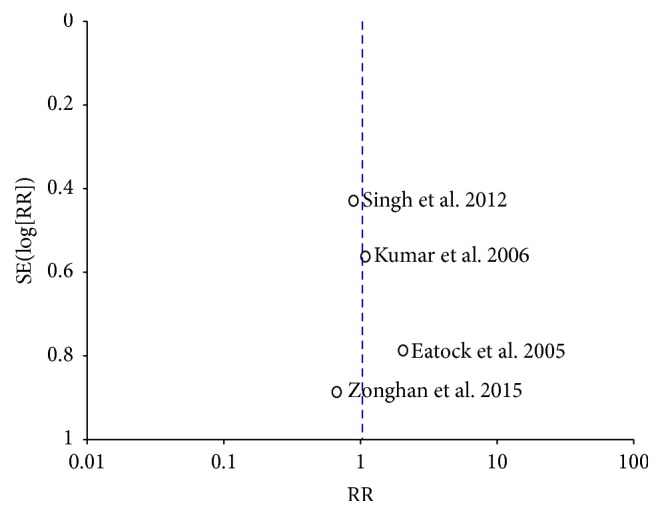
Funnel plot for publication bias.

No publication bias was observed based on a visual inspection of the funnel plot (Figure 8).

We planned to analyze other variables, such as the type and size of the tubes, placement complications, and placement methods of both tubes. However, the included studies did not sufficiently report this aspect or did not study this aspect. Therefore, it was difficult to perform this analysis on these variables [27].

## 4. Discussion

The treatment of SAP has gradually changed from early surgical treatment to conservative treatment for those patients who do not have a pancreatic infection [28]. However, the prognosis of SAP is still poor. EN is not only used as a way to provide nutritional support but also used as a measure to prevent infection. However, there is controversy regarding the route of EN. Previous meta-analyses [6, 21] showed that there were no significant differences in the safety and tolerance of NG and NJ nutrition in patients with SAP. There was no increase in mortality or adverse nutrition-associated events. Based on these results, the Guidelines of the American College of Gastroenterology (ACG) for the management of AP indicate that the efficacy and safety of NG nutrition and NJ nutrition are similar. However, the criteria used to classify SAP in the previous studies indicated above did not use the Atlanta 2012 classification criteria, and some patients with moderately SAP may be considered SAP. As a matter of fact, some controversial therapies may only show efficacy in more critically ill patients. Our up-to-date meta-analysis demonstrated that there were no significant differences in the mortality, infectious complications, digestive complications, achieving energy balance, and length of hospital stay between patients with SAP who received NG or NJ nutrition. Our results were similar to previous meta-analyses [6, 21].

Furthermore, there were many advantages for providing NG nutrition to patients with SAP because it was easier and cheaper to insert NG tubes. In the study by Eatock et al. [19], the NJ tubes were inserted using an endoscopic technique. In the study by Singh et al. [20], the NJ tube was also inserted under endoscopic guidance. In some other studies, the NJ tubes were placed using a fluoroscopy technique. Although both the endoscopy and fluoroscopy techniques are highly effective for placing NJ nutrition tubes, these techniques also have some disadvantages. Most hospital centers cannot perform the fluoroscopic procedure at the bedside. Therefore, the patient must be transported to the radiology site. Intrahospital transportation of critically ill patients is associated with up to 70% of adverse effects [6]. In addition, fluoroscopy exposes the patients and medical personnel to radiation. The techniques used in the endoscopic method are often complicated and require a significant learning curve. Therefore, under these conditions, the placement of the NJ tube is expensive and inconvenient compared to the placement of the NG tube, and NG nutrition is preferred for these patients.

Recently, Hu et al. [29] reported that metoclopramide and domperidone improve the postpyloric placement of NJ tubes in critically ill patients at the bedside; the success rates of postpyloric placement after 24 hours in the metoclopramide and domperidone groups were 55.0% and 51.5%, respectively. These drugs can allow patients to avoid or reduce the chance of endoscopy or fluoroscopy and make the placement of the NJ tube more convenient. Tube placement may affect the results of the comparison between NG and NJ nutrition in patients with SAP. We planned to analyze the methods used to place both tubes. However, the included studies did not sufficiently report this aspect or did not study this aspect. Therefore, it was difficult to perform the analysis on this variable [27], and further studies on this variable may be interesting.

Our meta-analysis included previous studies, and the criteria used to diagnose SAP in these studies were based on previous criteria that may have included many patients with moderately SAP. In the study by Eatock et al. [19], the total hospital stay was only 16 days (range 10–22 days) in the NG nutrition group and 15 days (range 10–42 days) in the NJ nutrition group. Moreover, only 26% of patients in the NG group and 36% of patients in the NJ group were transferred to the intensive care unit. It seemed that these patients did not have a particularly serious disease. In the study by Singh et al. [20], the total hospital stay in both groups was similar, namely, 17 days (range 1–73 days) in the NG group and 18 days (range 4–54 days) in the NJ group. In particular, only a few patients from both groups died 24 hours after they were enrolled in the study (only 4 and 7 patients died in the NG and NJ groups, resp.). It demonstrated that these patients may mainly exhibit moderately SAP. It is not clear whether the result will be the same using the new classification criteria.

There are many limitations in our meta-analysis. First, there were few studies that compared NG and NJ nutrition in patients with SAP, and the sample size included in our meta-analysis was small. Second, the studies included in our meta-analysis were all single center studies, and the external validity was limited. Third, because only 4 studies were included in our meta-analysis, an assessment of publication bias using a funnel plot will not provide sufficient power to reveal asymmetry. The capacity of funnel plots to detect bias is limited when the meta-analyses are based on a limited number of small trials. Additional studies are required to confirm our results. A large multicenter trial sponsored by the National Institutes of Health (NIH) is currently being performed to determine whether NG nutrition or NJ nutrition are better for patients with SAP [5]. We are awaiting the results.

## 5. Conclusions

Our meta-analysis demonstrated that there were no significant differences in the mortality, infectious complications, digestive complications, achieving energy balance, or length of hospital stay of patients with SAP who received NG or NJ nutrition. Further studies are required to confirm the results due to the limitations of our meta-analysis.

## Supplementary Material

Supplementary Material provides "PRISMA 2009 checklist."

## Figures and Tables

**Table 1:** Characteristics and quality of the included RCTs.

Study	Year	Country	Design	Sample size	Double blind	Withdrawals/dropouts (NG/NJ)	Randomization	Jadad score
Eatock et al. [19]	2005	UK	RCT	NG (27) NJ (22)	No	0/1	Yes	3

Kumar et al. [18]	2006	India	RCT	NG (16) NJ (14)	No	0/1	Yes	3

Singh et al. [20]	2012	India	RCT	NG (39) NJ (39)	No	0/0	Yes	3

Zonghan et al. [25]	2015	China	RCT	NG (40) NJ (40)	No	0/0	Yes	3

The modified Jadad score was used to evaluate the quality of the included trials.

NG, nasogastric nutrition; NJ, nasojejunal nutrition.

**Table 2:** Basic demographic characteristics of the patients in the included studies.

Study	Group	Number of patients	Age (years)	Gender (M/F)	Etiology		
					Gallstones	Alcohol	Idiopathic
Eatock et al., 2005 [19]	NG	27	63^△^ (47–74)	14/13	16	6	3
	NJ	22	58^△^ (48–64)	12/10	16	6	0

Kumar et al., 2006 [18]	NG	16	43.25^*∗*^ (12.76)	14/2	7	4	4
	NJ	14	35.57^*∗*^ (12.53)	11/3	4	4	5

Singh et al., 2012 [20]	NG	39	39.1^*∗*^ (16.7)	28/11	12	12	9
	NJ	39	39.7^*∗*^ (12.3)	25/14	21	10	7

Zonghan et al., 2015 [25]	NG	40	41^*∗*^ (25–60)	23/17	13	20	0
	NJ	40	43 (23–65)	22/18	12	20	0

^△^The values are presented as medians (range). ^*∗*^The values are presented as the mean ± standard deviations.

## List of Abbreviations

SAP: Severe acute pancreatitis
PN: Parenteral nutrition
EN: Enteral nutrition
NG: Nasogastric
NJ: Nasojejunal
RR: Risk ratio
ACG: American College of Gastroenterology
SMD: Standardized mean difference.
